# Resistance patterns of Escherichia coli causing urinary tract infection 

**Published:** 2015

**Authors:** Elaheh Ferdosi-Shahandashti, Mostafa Javanian, Masoomeh Moradian-Kouchaksaraei, Babak Yeganeh, Ali Bijani, Elahe Motevaseli, Fatemeh Moradian- Kouchaksaraei

**Affiliations:** 1 Department of Advanced Technology in Medicine (SATiM), Medical Biotechnology, Tehran University of Medical Sciences, Tehran, Iran.; 2 Infectious Diseases & Tropical Medicine Research Center, Babol University of Medical Sciences, Babol, Iran; 3 Department of Infectious Diseases, Babol University of Medical Sciences, Babol, Iran.; 4 Social Determinants of Health (SDH) Research Center, Babol University of Medical Sciences, Babol, Iran.; 5 Department of Advanced Technology in Medicine (SATiM), Molecular Medicine, Tehran University of Medical Sciences, Tehran, Iran.

**Keywords:** Urinary Tract Infection, UTI, Escherichia coli, Antibiotic susceptibility, Antibiotic resistance, Disk diffusion, Micro dilution

## Abstract

**Background:**

Urinary tract infection (UTI) is one of the most prevalent infectious diseases and Escherichia coli is its common cause. The aim of this study was to assess the resistance patterns of E.coli in urinary tract infections and to determine the susceptibility of E.coli to commonly used antimicrobials and also to evaluate the options for empirical treatment of UTI.

**Methods:**

This study was conducted in the Ayatollah Rouhani Teaching Hospital of Babol Medical Sciences University in North of Iran. Between January of 2013 to December 2013, antimicrobial susceptibility tests were done by disk diffusion and microdilution method. Growth of >_=_10^5^ cfu/ml was considered as positive urine test. Ten commonly used antibiotics were examined for susceptibility test. Data and the results were collected and analyzed.

**Results:**

E.coli grew in 57 urine samples. Imipenem, ofloxacin, ciprofloxacin were the most sensitive antibiotics at 87.7%, 87.7% and 78.9% respectively. Whereas, cotrimoxazole, cefexime, cefotaxcime and ceftriaxone were the most resistant antibiotics. Antibiotic sensitivity of disk diffusion compared minimum inhibitory concentration (MIC) detected by microdilution had the sensitivity, specificity, positive predictive value and negative predictive value of 82%, 98%, 99% and 74%, respectively.

**Conclusion:**

Imipenem, ofloxacin and ciprofloxacin should be used in empirical therapy of UTI.

Urinary tract infections (UTI) is the most common infection experienced by human after respiratory tract infection (1), accounting for 8.6 million visits (84% by women) in 2007 in the United States (2). Up to 60% of women have symptomatic UTIs during their lifespan, and 10% of women have UTIs annually. UTI in men is uncommon but often associated with structural abnormality (3). Although the most frequent etiology continues to be *Escherichia col*i (E.coli). Since the resistance patterns of E. coli strains causing UTI varies considerably between regions and countries, a specific treatment recommendation may not be universally suitable for all regions or countries (4). Active surveillance studies of in vitro susceptibility of uropathogens in women with uncomplicated cystitis are helpful in making decisions about empirical therapy (4). Evidence proves that antibiotic-resistance genes were present in the era before antibiotic therapy was available, and they probably originated from antibiotic-producing bacteria (5, 6). 

At least eight distinctive mechanisms of antibiotic resistance have been described in bacteria (7). To avoid treatment failure, and bacterial resistance, it is necessary to be aware of local antibiotic resistance for selecting an appropriate antibiotic for empiric therapy. The aim of this study was to assess the resistance patterns of E.coli causing urinary tract infections to commonly used antimicrobials and to evaluate the options for empirical treatment of UTI in Babol, North of Iran.

## Methods

This cross- sectional study was conducted in Ayatollah Rouhani Teaching Hospital of Babol Medical Sciences University, North of Iran, during 2013. Urine was collected from 57 community patients who referred to the hospital laboratory of the hospital. All urine samples collected by midstream clean-catch or catheterization were obtained. These samples were processed on blood agar and EMB medium with a standard loop and were incubated at 37^o^c for 24 hours.

Urine specimen positive for E.coli >_= _10^5^ cfu/ml referred to Department of Microbiology of Babol Medical School for confirmation and detection of susceptibility to antibiotics. Antibiotics susceptibility testing was performed by disk diffusion and microdilution method according to Clinical and Laboratory Standards Institute (CLSI) 2013 for ceftizoxime, cefotaxime, ceftriaxone, cefepime, cefexime, ciprofloxacin, ofloxacin, imipenem, cotrimoxazole and gentamicyn. 

To determine the susceptibility of bacteria to antibiotic sensitivity tests, antibiogram method was implemented using the disc initially sterile swab impregnated with microbial suspension Mueller Hinton agar medium. Then, by using forceps, antibiotic disks at least 1cm of each other in medium was placed. For each period forceps should be sterilized with a flame. After the disks, plates were incubated for 24h at 37 ° C. After 24 hours, the diameter of the inhibition zone around the disk was measured using a ruler and with CLSI 2013 as a resistant, semi-sensitive or sensitive bacteria had been reported.

To determine the MIC of the antibiotic microdilution, broth method was used. To provide appropriate dose of antibiotics, 5,000 micrograms of antibiotics were mixed in 500 microliter water soluble and allocated in1 microliter microtubes and then put them in the freezer. In microdilution broth method, each microplate has 96-wells. It had 8 rows and 12 columns. (The number of rows A-H and columns 1-12 were numbered). Blank column No. 11 as a negative control and column No. 12 a positive control.

At first in all wells of column 1 and the first well of negative control, 200 microliter of medium BHI broth was added. Then in the other wells 100 μl BHI broth medium was added. 

Then antibiotics were added in all wells of the first column and in the first well of negative control column. For the preparation of the antibiotic concentration ratio of ⅓, 100 ml of column 1 was transferred to column 2 and from column 2 to column 3 and to column 10 and 100 microliter remaining at the sampler top were discarded. Thus, the respective dose of antibiotics for columns 1 to 10 were prepared as follows: 128 μgr / ml ,64μ gr / ml, 32 μ gr / ml, 16 gr / ml, 8 μ gr / ml, 4 μ gr / ml , 2 μ gr / ml, 1 μ gr / ml, 0.5 μ gr / ml, 0. 25 μ gr / ml.

To prepare the appropriate concentration of antibiotics in negative control wells, 100 ml from well A transfered to well B and H, and the remaining 100 microliter at the tip of sampler was discarded. After bacteria were cultured on BHI medium, a half McFarland turbidity suspension was prepared.

The next phase of bacterial suspension was to create a dilution of 1/10 and then 5 microliter was added to all wells except the negative control well. Ultimately, the final volume of 100 ml was all wells. Negative control was free of bacteria and positive control was free of antibiotics. The microplate was incubated for 24 h and temperature was at 37°C. 

After 24 hours, the microplate was examined under light of which the last staining was observed as the minimum inhibitory concentrations of antibiotics that considered avulsion compared with CLSI 2013 table as resistant, moderately susceptible and susceptible of bacteria to antibiotics was reported.

In this evaluation, ceftizoxime, cefotaxime, ceftriaxone, cefepime, cefexime, ciprofloxacin, ofloxacin, imipenem, Co-trimoxazole and gentamicyn were studied. All agents were from Merck and Co. (Germany). 

Statical analysis was performed using the SPSS Version 17. The table ***making a CAT diagnosis*** was used to compare the disk diffusion with MIC, accumulation, positive predictive value, negative predictive value, sensitivity and specificity of disk diffusion.

## Results

In this study, we found that 57 E.coli isolated in our hospital were susceptible to imipenem, ofloxacin, ciprofloxacin with the following resistance of 87.7%, 87.7% and 78.9% respectively. Also, cotrimoxazole, cefexime, cefotaxime and ceftriaxone were the most resistant antibiotics (table 1). According to disk diffusion, ofloxacin, ciprofloxacin, and cefepime were the most sensitive antibiotics at 84.2%, 68.4% and 68.4%, respectively, whereas cotrimoxazole, ceftotaxime and ceftriaxone were the most resistant antibiotics (table 2).

Disk diffusion antibiotic sensitivity compared with MIC detection microdilution method had sensitivity, specificity, positive predictive value and negative predictive value of 82%, 98%, 99% and 74% respectively. 

**Table 1:** Antibiotics susceptibility of E.coli causing UTI by microdilution method

**Antibiotics **	**sensitive**	**Intermediate**	**resistance**
Ceftizoxime	66.7	7	27.3
Cefotaxime	56.1	8.8	35.1
Ceftriaxone	57.9	7	35.1
Ciprofloxacin	78.9	7	14
Ofloxacin	87.7	5.3	7
Imipenem	87.7	7	5.3
Co.trimoxazole	29.8	7	63.2
Cefepime	77.2	12.3	10.5
Cefixime	61.4	3.5	35.1
Gentamicin	63.2	12.3	24.6

**Table 2:** Antibiotics susceptibility of E.coli causing UTI by disk diffusion

**Antibiotics**	**sensitive**	**Intermediate**	**resistance**
Ceftizoxime	56.1	3.5	40.4
Cefotaxime	45.6	8.8	45.6
Ceftriaxone	52.6	1.8	45.6
Ciprofloxacin	68.4	7	24.6
Ofloxacin	84.2	7	8.8
Imipenem	56.1	5.3	38.6
Co.trimoxazole	24.6	10.5	64.9
Cefepime	68.4	8.8	22.8
Cefixime	50.9	5.3	43.9
Gentamicin	49.1	14	36.8

## Discussion

In this study, we found that the most isolated E.coli were susceptible to imipenem, ofloxacin and ciprofloxacin. These two oral agents can be used for outpatient cases, but those who were hospitalized should be treated with imipenem as parenteral agent. Several studies performed in Iran and other country had findings similar to the results of our study (8-13), but in contrast, in Spain and Germany, the susceptibility to ciprofloxacin was more than 90% (14, 15). In other studies performed in developing countries showed lower sensitivity to ciprofloxacin between 15% to 43.2% (16, 17). 

Another finding in our study was the high rate of resistance to cotrimoxazole as shown in other studies in other parts of Iran (10, 11, 13, 18) and Nigeria (16). But resistance to cotrimoxazole was low in Spain and Austria (14, 19). These differences may be due to the lower administration of this agent in these developed countries. Therefore, cotrimoxazole should not be used in developing countries as empirical therapy for UTI. In this study, we found moderate resistance to cefexime and other third generation cephalosporins as shown by other researchers in other cities in Iran (10-13, 18, 20). On the contrary, the resistance to cefotaxime and other third generation cephalosporins was low in Austria (19). These findings emphasize to delineate an algorithm to specify a treatment of patient in developed and developing countries. Another finding in our study was the similarity of the disk diffusion test and MIC for the detection of sensitivity of various antibiotics as investigated and approved by other researchers (21-23). The weakness of our study is the lack of information about the previous used antibiotic in our patients before entering in the study. In conclusion, our results showed that imipenem, ofloxacin and ciprofloxacin should be used in empirical therapy of UTI. Cotrimoxazole and third generation cephalosporins should not be used in empirical therapy of UTI especially pyelonephritis.

## Notes

**Funding:** This study was supported financially by the Infectious Diseases and Tropical Medicine Research Center of Babol University of Medical Sciences (grant No: 2106).

## References

[1] Longo DL (2012). Harrison's principles of internal medicine.

[2] Hooton TM (2012). Clinical practice. Uncomplicated urinary tract infection. N Engl J Med.

[3] Nicolle LE (2008). Uncomplicated urinary tract infection in adults including uncomplicated pyelonephritis. Urol Clin North Am.

[4] Gupta K, Hooton TM, Naber KG (2011). International clinical practice guidelines for the treatment of acute uncomplicated cystitis and pyelonephritis in women: a 2010 update by the Infectious Diseases Society of America and the European Society for Microbiology and Infectious Diseases. Clin Infect Dis.

[5] Medeiros AA (1997). Relapsing infection due to enterobacter species: lessons of heterogeneity. Clin Infect Dis.

[6] Gardner P, Smith DH, Beer H, Moellering RC (1969). Recovery of resistance (R) factors from a drug-free community. Lancet.

[7] Hawkey P (2008). Molecular epidemiology of clinically significant antibiotic resistance genes. Br J Pharmacol.

[8] Adjei O, Opoku C (2004). Urinary tract infections in African infants. Int J Antimicrob Agents.

[9] Yüksel S, Öztürk B, Kavaz A (2006). Antibiotic resistance of urinary tract pathogens and evaluation of empirical treatment in Turkish children with urinary tract infections. Int J Antimicrob Agents.

[10] Madani SH, Khazaee S, Kanani M, Shahi M (2008). Antibiotic resistance pattern of E. coli isolated from urine culture in Imam Reza Hospital Kermanshah-2006.. J Kermanshah Univ Med Sci.

[11] Molla Abaszadeh H, Haji Sheikhzadeh B (2014). Surveying the contamination rate, sensibility and antimicrobial resistance patterns in staphylococcus aureus isolated from traditional cheese consumed in Qotur of khoy province. J Fasa Univ Med Sci.

[12] Alós JI, Serrano MG, Gómez‐Garcés JL, Perianes J (2005). Antibiotic resistance of Escherichia coli from community‐acquired urinary tract infections in relation to demographic and clinical data. Clin Microbiol Infect.

[13] Safdari H, Ghazvini K (2007). Antimicrobial susceptibility patterns among E. Coli isolated from urinary tract infections in Ghaem University hospital, Mashhad.. Zahedan J Res Med Sci.

[14] Hamid-Farahani R, Tajik A, Noorifard M (2012). Antibiotic resistance pattern of E. coli isolated from urine culture in 660 Army clinical laboratory center in Tehran 2008. J Army Univ Med Sci.

[15] Schmiemann G, Gágyor I, Hummers-Pradier E, Bleidorn J (2012). Resistance profiles of urinary tract infections in general practice-an observational study. BMC Urol.

[16] Aboderin OA, Abdu AR, Odetoyin BW, Lamikanra A (2009). Antimicrobial Resistance in Escherichia coli Strains From Urinary Tract Infections. J Natl Med Assoc.

[17] Lu PL, Liu YC, Toh HS (2012). Epidemiology and antimicrobial susceptibility profiles of Gram-negative bacteria causing urinary tract infections in the Asia-Pacific region: 2009–2010 results from the Study for Monitoring Antimicrobial Resistance Trends (SMART). Int J Antimicrob Agents.

[18] Savadkouhi R, Sorkhi H, Pournasrollah M (2008). Antibiotic resistance of bacteria causing urinary tract infections in hospitalized patients in the pediatric subspecialty Hospital of Amir Kala, Babol, 2002-2005. Iran J Infect Dis Trop Med..

[19] Kamenski G, Wagner G, Zehetmayer S (2012). Antibacterial resistances in uncomplicated urinary tract infections in women: ECO· SENS II data from primary health care in Austria. BMC Infect Dis.

[20] Rajabnia-Chenari M, Gooran SH, Fazeli F, Dashipour AR (2012). Antibiotic resistance pattern in urinary tract infections in Imam-Ali hospital, Zahedan (2010-2011). Zahedan J Res Med Sci.

[21] Erfani Y, Rasti A, Mirsalehian A, Mirafshar S, Ownegh V (2011). E-test versus disk diffusion method in determining multidrug resistant strains of Escherichia coli in urinary tract infection. Afr J Microbiol Res.

[22] Erfani Y, Choobineh H, Safdari H, Rasti A, Alizadeh S (2008). Comparison of E. teat and disk diffusion agar in antibiotic susceptibility of. E. coli isolated from patients with urinary tract infection in Shariati hospital (Iran). Res J Biol Sci.

[23] Khalili H, Soltani R, Negahban S, Abdollahi A, Gholami K ( 2012). Reliability of disk diffusion test results for the antimicrobial susceptibility testing of nosocomial gram-positive microorganisms: is e-test method better?. Iran J Pharm Res.

